# *CURATE*: Scaling-Up Differentially Private Causal Graph Discovery

**DOI:** 10.3390/e26110946

**Published:** 2024-11-05

**Authors:** Payel Bhattacharjee, Ravi Tandon

**Affiliations:** Department of Electrical and Computer Engineering, University of Arizona, Tucson, AZ 85721, USA; tandonr@arizona.edu

**Keywords:** differential privacy, causal graph discovery, adaptive privacy budgeting

## Abstract

Causal graph discovery (CGD) is the process of estimating the underlying probabilistic graphical model that represents the joint distribution of features of a dataset. CGD algorithms are broadly classified into two categories: (i) constraint-based algorithms, where the outcome depends on conditional independence (CI) tests, and (ii) score-based algorithms, where the outcome depends on optimized score function. Because sensitive features of observational data are prone to privacy leakage, differential privacy (DP) has been adopted to ensure user privacy in CGD. Adding the same amount of noise in this sequential-type estimation process affects the predictive performance of algorithms. Initial CI tests in constraint-based algorithms and later iterations of the optimization process of score-based algorithms are crucial; thus, they need to be more accurate and less noisy. Based on this key observation, we present *CURATE* (CaUsal gRaph AdapTivE privacy), a DP-CGD framework with adaptive privacy budgeting. In contrast to existing DP-CGD algorithms with uniform privacy budgeting across all iterations, *CURATE* allows for adaptive privacy budgeting by minimizing error probability (constraint-based), maximizing iterations of the optimization problem (score-based) while keeping the cumulative leakage bounded. To validate our framework, we present a comprehensive set of experiments on several datasets and show that *CURATE* achieves higher utility compared to existing DP-CGD algorithms with less privacy leakage.

## 1. Introduction

Causal Graph Discovery (CGD) enables estimation of the partially connected directed acyclic graph (DAG) that represents the underlying joint probability distribution of the features of an observational dataset. CGD is an important part of causal inference [1] and is widely used in various disciplines, including biology [2], genetics [3], drug discovery, ecology [4], curriculum design [5], finance, and banking [6].

***Overview of Causal Graph Discovery (CGD):*** The process of estimating the causal graph from observational data relies on the execution of causal graph discovery algorithms. CGD algorithms are broadly classified into two categories: *constraint-based algorithms* and *score-based algorithms*. Constraint-based algorithms, including the PC algorithm (named after the authors Peter and Clark) [1], Fast Causal Inference (FCI) algorithm [7], and their variants [8], estimate the causal graph in two phases: first, in the *skeleton phase*, the algorithm starts with a fully connected graph, then updates the graph based on statistical conditional independence (CI) test results and returns a partially-connected undirected graph. To determine conditional independence, a variety of test statistics can be used, such as the *G-test* [9] or $\chi^{2}$-*test* [10], as well as correlation coefficients such as *Kendall’s Tau* [11] or *Spearman’s Rho* [12]. In the second *orientation phase*, the algorithm orients the undirected edges based on the CI test results obtained in the skeleton phase and returns the estimated causal graph. Constraint-based algorithms are theoretically guaranteed to converge to the complete partial directed acyclic graph (CPDAG) under certain conditions, including the correctness of the CI tests, causal sufficiency, Markov assumptions, etc. On the other hand, score-based algorithms estimate the causal graph from observational datasets by optimizing a score function. The algorithm assigns relevance scores such as the Bayesian Dirichlet equivalent uniform (BDe(u)) score [13], Bayesian Gaussian equivalent (BGe) score [14], Bayesian information criterion (BIC [15]), and minimum description length (MDL) [16] to all potential candidate graphs derived from the dataset and uses them to estimate the best graph. This method enables score-based algorithms to avoid the need for a large amount of CI tests. In a recent work, NOTEARS [17] converted the traditional combinatorial problem to a continuous optimization problem in order to estimate the DAG. However, these algorithms are computationally more expensive, as they must enumerate and score each and every conceivable graph among all of the variables provided.

***Privacy Threats and Differentially Private CGD:*** CGD algorithms often work with real-world datasets that may contain sensitive and private information about participants, including social and demographic details, credit histories, medical conditions, etc. Thus, releasing the causal graph itself or the intermediate statistical conditional independence (CI) test results may lead ot privacy leakage. Recent work [18] has demonstrated *membership inference threats* through probabilistic graphical models. Several recent works have adopted the notion of differential privacy (DP) [19] in the context of CGD to ensure a certain level of user privacy.

**Figure 1:**
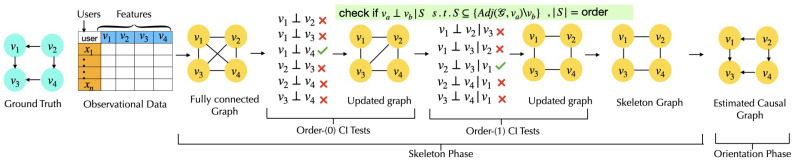
Generic workflow of constraint-based CGD algorithms, showing the skeleton orientation phases. The skeleton phase starts with a fully connected graph consisting of *d* nodes, where *d* is the number of features/variables and $k_{i}$ is the maximum number of CI tests in order *i*. The sequence and number of tests in any order *i* are dependent on the outcomes of the order (i−1) tests. Notably, the skeleton phase is prone to privacy leakage.

For instance, existing constraint-based differentially private CGD (DP-CGD) algorithms incorporate several differential privacy techniques to perturb the CI test statistic, including the *Laplace Mechanism* (PrivPC) [20], *Exponential Mechanism* (EM-PC) [21], and *Sparse Vector Technique* (SVT-PC) [20]. For score-based algorithms, NOLEAKS [22] adopted the *Gaussian Mechanism* to perturb the gradient of the optimization problem. However, existing algorithms rely on the method of adding the *same amount of noise* to each iteration of the estimation process. As shown in Figure 1 and discussed in Section 3, the CI tests in constraint-based CGD can be highly interdependent. If an edge between two variables is deleted by a CI test, then the conditional interdependence (conditioned on any other subset of features) is never checked in later iterations. Furthermore, this issue also impacts the scalability of private CGD, as the total privacy leakage blows up for datasets with a large number of features (d>>1). Meanwhile, differentially private score-based algorithms such as NOLEAKS [22] optimize the objective function to obtain the adjacency matrix of the estimated DAG. Because this optimization technique utilizes noisy gradients of the objective function, adding the same amount of noise may lead to higher convergence times, as the optimal point may be missed by the algorithm during noise addition. To prevent the algorithm from missing optima and speed up converge, the later iterations of the optimization process should ideally be less noisy.

***Overview of the Proposed CURATE Framework:*** The aforementioned observations bring forth the important point of adaptive privacy budgeting for both constraint-based and score-based differentially private CGD algorithms. For constraint-based algorithms, the initial CI tests are more crucial, as decisions regarding edge deletion are never checked in the later iterations. Within the scope of score-based algorithms, the later iterations in optimization are more critical. This motivates the idea of *adaptive privacy budgeting*. Given a total privacy budget, such budgeting can reduce the risk of errors propagating to subsequent iterations and improve the scalability of constraint-based algorithms. On the other hand, score-based algorithms ideally have less noise and more accuracy in the later iterations. Intuitively, allocating a higher privacy budget to later iterations of the optimization process should help to reduce the risk of missing the optima of the objective function. In this paper, we present an adaptive privacy budgeting framework called *CURATE* (CaUsal gRaph AdapTivE privacy) for both constraint- and score-based CGD algorithms in differentially private environments. The main contributions of this paper are summarized as follows:

- Our proposed *CURATE* framework scales up the utility of the CGD process using adaptive privacy budget allocation. Within the scope of constraint-based DP-CGD algorithms, the constraint-based *CURATE* algorithm optimizes privacy budgets for each order of CI test (CI tests of the same order have the same privacy budget) in a principled manner, with the goal of minimizing the surrogate for the total probability of error. By allocating adaptive (and often comparatively higher) privacy budgets to the initial CI tests, *CURATE* ensures better overall predictive performance with less total leakage compared to the existing constraint-based DP-CGD algorithms.
- We present a score-based *CURATE* algorithm which allows for adaptive budgeting to maximize the number of iterations given a fixed privacy budget ($\epsilon_{\mathrm{Total}}$). The score-based *CURATE* algorithm uses a functional causal model-based optimization approach that allocates a higher privacy budget to later iterations. The privacy budget is incremented as a function of iterations, helping our score-based *CURATE* to achieve better utility in comparison to existing works.
- We present extensive experimental results on six public CGD datasets to compare the predictive performance of our proposed *CURATE* framework with existing DP-CGD algorithms. Our experimental results show that *CURATE* ensures better predictive performance with less leakage by orders of magnitude. The average required number of CI tests in constraint-based *CURATE* is also significantly less than that of existing constraint-based DP-CGD algorithms.

## 2. Preliminaries on CGD and DP

In this section, we review the notion of causal graph discovery and provide a brief overview of both constraint-based algorithms (canonical PC) and FCM-based algorithms (NOTEARS, NOLEAKS) along with the description of differential privacy [19,23].

**Definition** **1** (Probabilistic Graphical Model)**.** Given a joint probability distribution $P(F_{1},\ldots ,F_{d})$ of d random variables, the graphical model $G^{*}$ with V vertices ($v_{1},\ldots ,v_{d}$) and E⊆V×V edges is known as a Probabilistic Graphical Model (PGM) if the joint distribution decomposes as

$$\begin{array}{c} P\left(F_{1},\ldots ,F_{d}\right)=\prod_{F_{a}\in \left\{F_{1},\ldots ,F_{d}\right\}}P\left(F_{a}|Pa\left(F_{a}\right)\right), \end{array}$$

*where $Pa(F_{a})$ represents the direct parents of the node $F_{a}$. A PGM relies on the assumption that probabilistic independence (Fa⫫Fbp|S) ⇒ graphical independence (va⫫vbG|S) [24].*

**Definition** **2** (Causal Graph Discovery)**.** Given a dataset D with a collection of n i.i.d. samples $\left(x_{1},\ldots ,x_{n}\right)$ drawn from a joint probability distribution $P(F_{1},\ldots ,F_{d})$, where $x_{i}$ is a d-dimensional vector representing the d features/variables of the $i^{th}$ sample (user), the method of estimating the PGM $\left(G^{*}\right)$ from D is known as causal graph discovery (CGD) [20].

**Definition** **3** (ϵ,δ)-Differential Privacy [19,23,25])**.** *For all pairs of neighboring datasets D and $D^{\prime }$ that differ by a single element, i.e., $||D-D^{\prime }{\left|\right|}_{1}\leq 1$, a randomized algorithm M with an input domain of D and output range R is considered to be (ϵ,δ)-differentially private if ∀S⊆R:*

$$\begin{array}{c} P\left[M\left(D\right)\in S\right]\leq e^{\epsilon }P\left[M\left(D^{\prime }\right)\in S\right]+\delta . \end{array}$$

Differentially private CGD algorithms have adopted the *exponential mechanism* [21], *Laplace mechanism, sparse vector technique* [20], and *Gaussian mechanism* [22] to ensure DP.

**Definition** **4** ($l_{k}$-Sensitivity)**.** *For two neighboring datasets D and $D^{\prime }$, the $l_{k}$-sensitivity of a function f(·) is defined as*

$$\begin{array}{c} \Delta_{k}\left(f\right)=\max_{D,D^{\prime }\in R,\left|D,D^{\prime }\right|\leq 1}||f\left(D\right)-f\left(D^{\prime }\right){\left|\right|}_{k}. \end{array}$$

For instance, the Laplace mechanism perturbs the CI test statistic f(·) with Laplace noise proportional to the $l_{1}$-sensitivity of the function f(·), whereas the Gaussian mechanism adds noise proportional to the $l_{2}$-sensitivity to guarantee DP. Ideally, the classical Gaussian mechanism uses ϵ≤1 for (ϵ,δ) DP guarantees; however, this condition may not be sufficient in all CGD scenarios [22]. Therefore, the score-based DP algorithm in [22] uses the analytical Gaussian mechanism [26].

**Definition** **5** (Analytical Gaussian Mechanism [26])**.** *For a function $f:X\leftarrow R^{d}$ with $l_{2}$-sensitivity $\Delta_{2}$ and privacy parameters ϵ≥0 and δ∈[0,1], the Gaussian output perturbation mechanism A(x)=f(x)+Z with $Z\;N(0,\sigma^{2}I)$ is (ϵ,δ)-DP if and only if*

(1) $$\Phi \left(\frac{\Delta_{2}}{2\sigma }-\frac{\epsilon \sigma }{\Delta_{2}}\right)-e^{\epsilon }\Phi \left(\frac{-\Delta_{2}}{2\sigma }-\frac{\epsilon \sigma }{\Delta_{2}}\right)\leq \delta ,$$

*where *Φ* is the CDF of the Gaussian distribution.*

***Overview of Constraint-Based Algorithms:*** Canonical constraint-based CGD algorithms such as the PC algorithm  [1] work in two phases: a *skeleton phase* followed by an *orientation phase*. In the *skeleton phase*, the algorithm starts with a fully connected graph (G) and prunes it by conducting a sequence of conditional independence (CI) tests. The CI tests in PC are order-dependent, and the order of a test represents the cardinality of the conditioning set *S* of features. In order-(i) tests, all connected node pairs ($v_{a},v_{b}$) in G are tested for statistical independence conditioned on the set *S*. The conditioning set *S* is chosen such that $S\subseteq \{Adj\left(G,v_{a}\right)\backslash v_{b}\}$, where Adj(G,v) represents the adjacent vertices of node *v* in graph G. The edge between the node pairs ($v_{a},v_{b}$) is deleted if they both pass the order-(i) CI test, after which no further testing is performed for statistical independence conditioned on set *S* with |S|>i. The remaining edges in G are then tested for independence in order-(i+1) CI tests conditioned on a set *S* with |S|=(i+1). This process of CI testing continues until all connected node pairs in G are tested by conditioning on set *S* of size (d−2). At the end of this phase, the PC algorithm returns the skeleton graph. Next, in the *orientation phase*, the algorithm orients the edges based on the separation set *S* of one independent node pair ($v_{a},v_{b}$) without introducing cyclicity in G [1,21], as shown in Figure 1. In this two-step process, privacy leakage only occurs in the *skeleton phase*, as this is when the algorithm directly interacts with the dataset D. Therefore, the existing literature focuses on effectively privatizing CI tests subject to the notion of differential privacy [19,23], which can ensure that the presence/absence of a user will not *significantly* change the estimated causal graph.

***Overview of Score-Based Algorithms:*** Score-based algorithms estimate the DAG that optimizes a predefined score function. Due to their combinatorial acyclicity constraints, learning DAGs from data is NP-hard [27]. To address this issue, the NOTEARS score-based CGD algorithm [17] proposes a continuous optimization problem with an acyclicity constraint to estimate the DAG from observational data, eliminating the need to search over the combinatorial space of DAGs. From a group of DAGs, the DAG is selected which optimizes a predefined score function score(·) while satisfying the acyclicity constraints. Given an observational dataset D with *n* i.i.d. samples and *d* features $F=(F_{1},F_{2},\ldots ,F_{d})$, the algorithm estimates (mimics) the data generation process $f_{i}\left(\cdot \right)$ for every *i*-th feature/variable by minimizing the loss function. Essentially, the adjacency matrix *W* that represents the edges of the graph G is modeled with the help of a functional causal model (FCM). FCM-based methods represent every *i*-th variable $F_{i}$ of the dataset D as a function of its parents $\mathrm{Pa}(F_{i})$, and represent the added noise *Z* as follows:

$$\begin{array}{c} F_{i}=f_{i}\left(\mathrm{Pa}\left(F_{i}\right)\right)+Z. \end{array}$$

The key idea behind FCM-based CGD is to estimate the weight vector $w_{i}$ for each variable $F_{i}$ given its parents $\mathrm{Pa}(F_{i})$. Therefore, each variable $F_{i}$ can be represented as a weighted combination of its parents and noise *Z* as $F_{i}=w_{i}^{T}F+Z$. The optimization process of estimating the weight vector $w_{i}$ is based on the idea of minimizing the squared loss function $\ell \left(W,D\right)=\frac{1}{2n}||D-{DW||}_{F}^{2}$, where *W* is the associated adjacency matrix of the dataset D and *n* is the number of samples. The loss function indicates how well the adjacency matrix *W* captures the dependencies (causal structure) in dataset D. The goal is to minimize the loss ℓ(W,D) in order to find the optimal adjacency matrix *W*. The squared loss ℓ(W,D) is defined over $R^{d\times d}$, while the minimizer of ℓ(W,D) recovers the true directed acyclic graph on finite samples with high probability [17]. In the optimization process, the algorithm also uses a penalty function ${\lambda ||W||}_{1}$ that penalizes dense graphs. The detailed working mechanism of FCM-based CGD algorithms is described in Section 3.

***Sensitivity Analysis and Composition of DP:*** For the class of constraint-based algorithms, an edge between nodes ($v_{a},v_{b}$) from the estimated graph G is deleted conditioned on set *S* if ($f_{v_{a},v_{b}|S}\left(D\right)>T$), where $f_{v_{a},v_{b}|S}\left(\cdot \right)$ is the test statistic and *T* is the test threshold. Thus, the structure of the estimated causal graph depends on the nature of f(·) and the threshold (*T*). Additionally, the amount of added noise in DP-CGD is proportional to the $l_{k}$-sensitivity ($\Delta_{k}$) of the test statistic $f_{v_{a},v_{b}|S}\left(\cdot \right)$. Therefore, to maximize the predictive performance, test statistics that have lower sensitivity with respect to sample size *n* are preferred. Through analysis, we have observed that the $l_{1}$-sensitivity of the *Kendall’s* τ test statistic can be bounded as $\Delta_{1}\leq \frac{C}{\sqrt{n}}$, where *C* is a constant obtained through the analysis presented in Appendix A.2). Notably, any of the other CI test statistics mentioned in Section 1 can be used in the constraint-based *CURATE* framework. The class of score-based algorithms focuses on optimizing a score function to estimate the causal graph. These algorithms often rely on gradient-based methods, with the gradient of the objective function frequently being clipped and perturbed to preserve privacy. As mentioned in [22], the $l_{2}$-sensitivity of the clipped gradient can be bounded as $\Delta_{2}\leq \frac{ds}{n}$, where *s* is the clipping threshold. In [22], the authors further exploited the properties of the dataset and adjacency matrix, allowing the $l_{2}$ of the gradient to be be assigned an upper bound of $\Delta_{2}\leq \frac{\sqrt{d(d-1)s}}{n}$. *Composition* is a critical tool in DP-CGD, since differentially private CGD algorithms discover the causal graph in an iterative process. Constraint-based CGD algorithms run a sequence of interdependent tests, while score-based algorithms optimize a predefined score function in an iterative manner. Therefore, the total privacy leakage can be calculated using *basic composition* [19,23,28,29], *advanced composition* [25,29], *optimal composition* [30], *adaptive composition* [31], or *moments accounting* [32]. Within the scope of this paper, we consider basic composition (also known as sequential composition) and advanced composition. Because each order-*i* conditional independence (CI) test in the constraint-based *CURATE* algorithm has the same privacy budget and failure probability, we apply advanced composition to calculate the per-order privacy leakage. However, as the constraint-based and score-based *CURATE* algorithms have different privacy budgets over different iterations (orders), we apply basic composition to calculate the total leakage.

## 3. Adaptive Differential Privacy in Causal Graph Discovery

This section presents the main idea of this paper, our *CURATE* adaptive privacy budgeting framework. In Section 3.1, we demonstrate the adaptive privacy budgeting mechanism for constraint-based algorithms. We introduce and explain the basic optimization problem that enables the allocation of the adaptive privacy budget through all the iterations (orders) of the CI tests. In Section 3.2, we present adaptive privacy budget allocation for score-based algorithms. We introduce adaptivity while ensuring differential privacy (DP) during evaluation of the weighted adjacency matrix. This section provides the theoretical foundation behind the adaptive privacy budget allocation mechanism in the context of DP-CGD.

### 3.1. Adaptive Privacy Budget Allocation with Constraint-Based *CURATE* Algorithm

**Figure 2:**
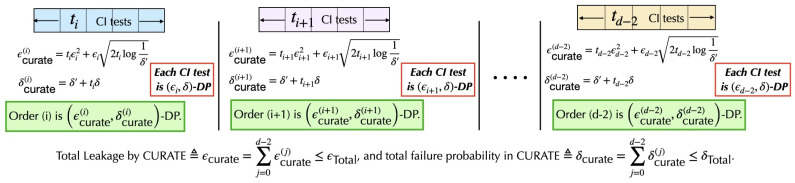
The composition mechanism in constraint-based *CURATE* across all orders of CI tests. For every order (i), the total privacy leakage is calculated with advanced composition, as the privacy budgets and failure probabilities for all order-(i) tests are the same. The total leakage across all orders is then calculated by constraint-based *CURATE* using basic composition.

In this section, we present *CURATE* for enabling adaptive privacy budgeting while minimizing the error probability. As the CI tests in constraint-based CGD algorithms are highly interdependent, predicting the total number of CI tests in CGD before the execution of the tests is difficult. The number of order-(i) CI tests $t_{i}$ enables the framework to approximate the per-order privacy budgets for later iterations $\epsilon_{i},\ldots ,\epsilon_{d-2}$ based on the total remaining privacy budget $\epsilon_{\mathrm{Total}}^{\left(i\right)}$. A naive data-agnostic way to choose an upper bound on $t_{i}$ is ti≤d2×d−2i, where d2 represents the number of ways to select an edge from the edges of a fully connected graph (the way of selecting an edge between two connected nodes out of *d* nodes) and d−2i refers to the selection of the conditioning set *S* with cardinality |S|=i. However, this upper bound is too large, and does not depend on the outcome of the previous iteration. A better approximation of $t_{i}$ is always possible given the outcome of the previous iteration. As DP is immune to *postprocessing* [23], releasing the number edges $e_{i+1}$ after executing order-(i) differentially private CI tests will preserve differential privacy. For instance, the possible number of order-(i+1) CI tests can always be upper-bounded as ti+1≤ei+1×d−2i+1, where $e_{i+1}$ represents the remaining edges after the order-(i) tests. After studying both methods, we have observed that ti+1≤ei+1×d−2i+1 is a better estimate of $t_{i+1}$, as ei≤d2,∀i∈{0,d−2}. Given the outcome of graph G of the order-(i−1) tests, with edges $e_{i}$ and a total (remaining) privacy budget of $\epsilon_{\mathrm{Total}}^{\left(i\right)}$, we assign privacy budgets $\left(\epsilon_{i},\ldots ,\epsilon_{d-2}\right)$. As every order-(i) CI test in *CURATE* is $\left(\epsilon_{i},\delta \right)$-DP with DP failure probability $\delta ,\delta^{\prime }>0$, the total leakage in order (i) is calculated with advanced composition [25] as $\epsilon_{\mathrm{curate}}^{\left(i\right)}=t_{i}\epsilon_{i}^{2}+\sqrt{2\log\left(\frac{1}{\delta^{\prime }}\right)t_{i}\epsilon_{i}^{2}}$, while the total failure probability in DP is calculated as $\delta_{curate}^{\left(i\right)}=\left(\delta^{\prime }+t_{i}\delta \right)$. However, because different orders have different privacy budgets, the total privacy leakage in *CURATE* is calculated through basic composition [25] as $\sum_{j=0}^{d-2}\epsilon_{\mathrm{curate}}^{\left(j\right)}=\sum_{j=0}^{d-2}\left(t_{j}\epsilon_{j}^{2}+\sqrt{2t_{j}\log\left(\frac{1}{\delta^{\prime }}\right)\epsilon_{j}^{2}}\right)$, while the cumulative failure probability of *CURATE* is $\sum_{j=0}^{d-2}\delta_{curate}^{\left(j\right)}$ (refer to Figure 2). Therefore, given the outcome of the order-(i−1) tests, the total leakage in *CURATE* must satisfy $\sum_{j=i}^{d-2}\left(t_{j}\epsilon_{j}^{2}+\sqrt{2t_{j}\log\left(\frac{1}{\delta^{\prime }}\right)\epsilon_{j}^{2}}\right)\leq \epsilon_{\mathrm{Total}}^{\left(i\right)}$, where tj=ej×d−2j and $\sum_{j=0}^{d-2}\delta_{curate}^{\left(j\right)}\leq \delta_{\mathrm{Total}}$. Moreover, we enforce $\epsilon_{i}\geq \epsilon_{i+1}\geq \ldots \geq \epsilon_{d-2}$ to ensure that the initial CI tests receive a higher privacy budget.

***DP-CI Test in CURATE***: The differentially private order-(i) CI test with privacy budget $\epsilon_{i}$ for variables $(v_{a},v_{b})\in G$ conditioned on a set of variables *S* is defined as follows:

- If $\hat{f}>T\left(1+\beta_{2}\right)\Rightarrow$ delete edge ($v_{a},v_{b}$)
- Else, if $\hat{f}<T\left(1-\beta_{1}\right)\Rightarrow$ keep edge ($v_{a},v_{b}$)
- Else, keep the edge with probability $\frac{1}{2}$

where $\hat{f}:=f_{v_{a},v_{b}|S}\left(D\right)+\mathrm{Lap}\left(\frac{\Delta }{\epsilon_{i}}\right)$, $\mathrm{Lap}(\frac{\Delta }{\epsilon_{i}})$ is the *Laplace noise*, Δ denotes the $l_{1}$-sensitivity of the test statistic, *T* denotes the threshold, and $\left(\beta_{1},\beta_{2}\right)$ denote the margins. In order to keep the utility high, we would ideally like to pick $\left(\epsilon_{i},\epsilon_{i+1},\cdots ,\epsilon_{d-2}\right)$ that minimizes the error probability $P\left[E\right]=P\left[G\neq G^{*}\right]$, where $G^{*}$ is the true causal graph and G is the estimated causal graph. Unfortunately, we do not have access to $G^{*}$; thus, in this paper we instead propose using a *surrogate* for the error by considering type-I and type-II errors relative to the unperturbed (non-private) statistic. A relative type-I error relative to the unperturbed CI test occurs when the private algorithm retains the edge and the unperturbed test statistic deletes the edge $\left(f_{v_{a},v_{b}|S}\left(D\right)>T\right)$, while a relative type-II error occurs when the algorithm deletes an edge and the unperturbed test statistic keeps that edge $\left(f_{v_{a},v_{b}|S}\left(D\right)<T\right)$. The next *Lemma* provides the upper bounds on the relative type-I and type-II error probabilities in *CURATE*.

**Lemma** **1.** *For some $c_{1},c_{2}\in \left(0,1\right)$ and non-negative test threshold margins $\left(\beta_{1},\beta_{2}\right)$, the relative type-I ($P[E_{1}^{i}]$) and type-II ($P[E_{2}^{i}]$) errors in order-(i) CI tests in CURATE with privacy budget $\epsilon_{i}$ and $l_{1}$-sensitivity *Δ* can be bounded as follows:*

$$\begin{array}{c} P\left[E_{1}^{i}\right]\leq \underset{q_{i}^{\left(1\right)}}{\underset{︸}{\frac{c_{1}}{2}+\frac{1}{2}e^{\left(-\frac{T\beta_{1}\epsilon_{i}}{\Delta }\right)}}},\;P\left[E_{2}^{i}\right]\leq \underset{q_{i}^{\left(2\right)}}{\underset{︸}{\frac{c_{2}}{2}+\frac{1}{2}e^{\left(-\frac{T\beta_{2}\epsilon_{i}}{\Delta }\right)}}}. \end{array}$$

**Figure 3:**
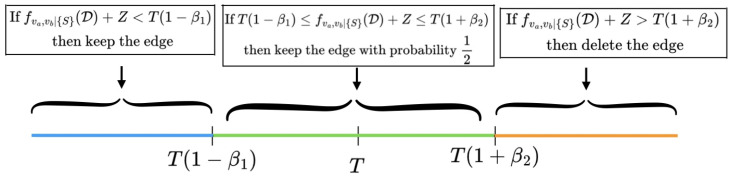
The threshold marginalization mechanism adopted in the constraint-based CURATE algorithm. The margins $\beta_{1},\beta_{2}$ allow for additional flexibility during hypothesis testing with noisy CI tests.

The proof of Lemma 1 is presented in the Appendix A. Because adding Laplace noise to the test statistic introduces randomness into the hypothesis testing process, we have designed threshold margins to precisely bound the error probabilities. As shown in Figure 3, we decide whether to retain or delete an edge based on the noisy test statistic $\hat{f}\left(\cdot \right)$. The margins $\beta_{1}$ and $\beta_{2}$ can also be optimized and adaptively chosen based on dataset characteristics. For instance, if the noisy test statistics lie far from the threshold *T*, a larger margin can be allowed and higher values for $\beta_{1}$ and $\beta_{2}$ can be selected; conversely, if the noisy test statistics are near the threshold, then it is necessary to reduce the margins in order to make better use of hypothesis testing. The main objective of *CURATE* is to adaptively allocate privacy budgets for order-(i) CI tests by minimizing the total relative error. The leakage in DP-CGD depends on the number of CI tests, and the number of CI tests depends in turn upon the number of edges in the estimated graph G. As the number of edges in the true graph is unknown, we use $P\left[E_{1}^{i}\right]+P\left[E_{2}^{i}\right]$ as a surrogate for the total error probability P[E]. Given the outcome of order-(i−1) tests, the algorithm can make a type-I error by preserving an edge that is not present in the true graph until order d−2. If such an edge is present after the order-(i−1) tests, the probability of a type-I error at the end of order d−2 can be represented as $\prod_{j=i}^{d-2}q_{j}^{\left(1\right)}$, as the addition of independent noise to each CI test enables the framework to bound the probability of error in each order independently, and the total error probability at the end of order (d−2) is the cumulative error made by the algorithm in every order (j). Similarly, the probability of keeping an edge which is present in the ground truth after order-(i−1) tests can be represented as $\prod_{j=i}^{d-2}\left(1-q_{j}^{\left(2\right)}\right)$; therefore, the total type-II error can be represented as $\left(1-\left(\prod_{j=i}^{d-2}\left(1-q_{j}^{\left(2\right)}\right)\right)\right)$. Given the outcome of order-(i−1) CI tests G, this allows us to construct the main objective function of this paper. The objective function that we propose to minimize is

(2) $$\begin{array}{c} \prod_{j=i}^{d-2}q_{j}^{\left(1\right)}+\left(1-\left(\prod_{j=i}^{d-2}\left(1-q_{j}^{\left(2\right)}\right)\right)\right). \end{array}$$

Because the number of edges in the true graph is unknown, we propose to minimize (2) as a surrogate for the error probability.

***Optimization for Privacy Budget Allocation:*** By observing the differentially private outcome of order-(i−1) CI tests (the remaining edges $e_{i}$ in graph G), *CURATE* optimizes for $\bar{\epsilon }=\left\{\epsilon_{i},..,\epsilon_{d-2}\right\}$ (the privacy budgets for subsequent order-(i) tests and beyond) while minimizing the objective function as described in (2). Formally, we define the following optimization problem in *CURATE*, denoted as $OPT(\epsilon_{\mathrm{Total}}^{\left(i\right)},e_{i},i)$:

(3) $$\underset{OPT(\epsilon_{\mathrm{Total}}^{\left(i\right)},e_{i},i)}{\underset{︸}{\arg\min_{\bar{\epsilon }}\prod_{j=i}^{d-2}q_{j}^{\left(1\right)}+\left(1-\left(\prod_{j=i}^{d-2}\left(1-q_{j}^{\left(2\right)}\right)\right)\right)}}\;s.t.\;\left\{\begin{array}{cc} \; & \sum_{j=i}^{d-2}\underset{\mathrm{total}\;\mathrm{leakage}\;\mathrm{in}\;\mathrm{order}-(j)}{\underset{︸}{\left(t_{j}\epsilon_{j}^{2}+\sqrt{2\log\left(\frac{1}{\delta^{\prime }}\right)t_{j}\epsilon_{j}^{2}}\right)}}\leq \epsilon_{\mathrm{Total}}^{\left(i\right)}\\ \; & \epsilon_{j}\geq \epsilon_{j+1}. \end{array}\right.$$

Given the outcome of order-(i−1) tests, the above optimization function $OPT(\epsilon_{\mathrm{Total}}^{\left(i\right)},e_{i},i)$ takes the following inputs: (a) the remaining total budget $\epsilon_{\mathrm{Total}}^{\left(i\right)}$, (b) the remaining edges $e_{i}$ in the output graph G after all order-(i−1) tests, and (c) the order index, i.e., order *i*. The function then optimizes and outputs the privacy budgets $\epsilon_{i},\ldots ,\epsilon_{d-2}$ for the remaining order tests while satisfying the two constraints mentioned in (3). Because the optimization problem in (3) is difficult to solve in a closed form, in our experiments we used sequential least squares programming (SLSQP) to optimize the objective function.

***Constraint-Based CURATE Algorithm:*** Next, we present the constraint-based *CURATE* as Algorithm 1, which enables adaptive privacy budget allocation for each order-*i* conditional independence test by solving the optimization problem in (3). The constraint-based algorithm in *CURATE* uses the optimization function OPT(·) recursively to adaptively observe chosen per-iteration privacy budgets. Given the total privacy budget $\epsilon_{\mathrm{Total}}^{\left(i\right)}$ for an order-*i* test, OPT(·) calculates the remaining privacy budget for order-(i+1) CI tests based on the number $t_{i}$ of order-*i* CI tests:

$$\begin{array}{c} \underset{\mathrm{budget}\;\mathrm{for}\;\mathrm{order}-(i+1)}{\underset{︸}{\epsilon_{\mathrm{Total}}^{\left(i+1\right)}}}=\underset{\mathrm{budget}\;\mathrm{for}\;\mathrm{order}-i\;}{\underset{︸}{\epsilon_{\mathrm{Total}}^{\left(i\right)}}}-\underset{\mathrm{actual}\;\mathrm{leakage}\;\mathrm{in}\;\mathrm{order}-i}{\underset{︸}{\left(t_{i}\epsilon_{i}^{2}+\epsilon_{i}\sqrt{2t_{i}\log\left(\frac{1}{\delta^{\prime }}\right)}\right)}}. \end{array}$$

Initially, the remaining budget for order-0 CI tests is equal to the assigned total privacy budget, i.e., $\epsilon_{\mathrm{Total}}^{\left(0\right)}=\epsilon_{\mathrm{Total}}$, and the edges in the complete graph $G_{0}$ can be expressed as e0=d2. In order 0, *CURATE* solves for $\epsilon_{0},\ldots ,\epsilon_{d-2}$ using the function $OPT(\epsilon_{\mathrm{Total}}^{\left(0\right)},e_{0},0)$. After completion of all order-0 CI tests, the algorithm calculates the remaining budget for order-1 CI tests as $\epsilon_{\mathrm{Total}}^{\left(1\right)}=\epsilon_{\mathrm{Total}}^{\left(0\right)}-\left(t_{0}\epsilon_{0}^{2}+\epsilon_{0}\sqrt{2t_{0}\log\left(\frac{1}{\delta^{\prime }}\right)}\right)$; by observing the remaining edges $e_{1}$, it then solves for the next set of privacy budgets $\epsilon_{1},\ldots ,\epsilon_{d-2}$. This process is then recursively applied for all i∈{0,1,…,d−2}, corresponding to all order-*i* tests.    
**Algorithm 1:**
 *Constraint-based CURATE Algorithm*

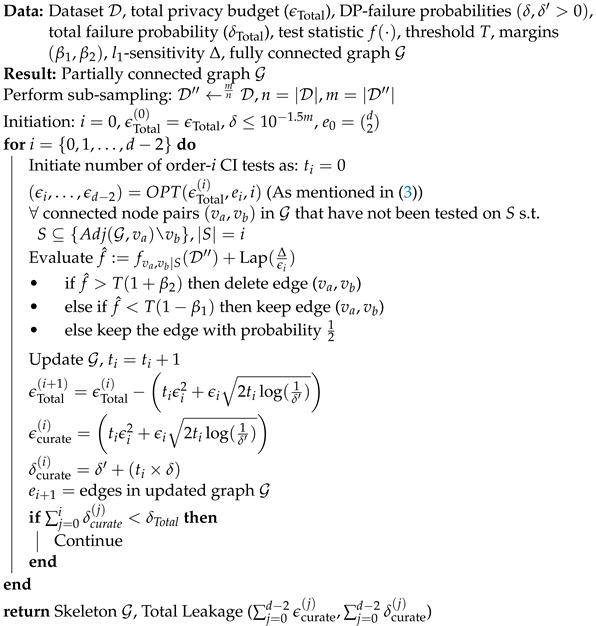


*Sub-sampling* has also been adopted by several recent works on DP-CGD [20,22]. As *sub-sampling* amplifies differential privacy [26], we can also readily incorporate sub-sampling parameters within the optimization frameworks of both constraint-based and score-based *CURATE*.

### 3.2. Adaptive Privacy Budget Allocation with Score-Based *CURATE* Algorithm

In this subsection, we present the adaptive and nonuniform privacy budget allocation mechanism for the class of score-based algorithms. This mechanism is based on the functional causal model (FCM) idea. Traditional score-based algorithms estimate the causal graph that optimizes a predefined score function, such as the Bayesian Dirichlet equivalent uniform (BDe(u)) score [13], Bayesian Gaussian equivalent (BGe) score [14], Bayesian information criterion (BIC) [15], or minimum description length (MDL) [16]. These methods are agnostic to the underlying true distribution of the data. There is a line of work in the literature that aims to extract more accurate underlying distributions from observational data through a functional causal model (FCM). Given an observational dataset D with $\left(x_{1},\ldots ,x_{n}\right)$ i.i.d. samples and *d* number of features ($F=\{F_{1},\ldots ,F_{d}\}$), FCM-based methods mimic the data generation process $f_{i}\left(\cdot \right)$ to obtain feature $F_{i}$ as a function of its parents $Pa(F_{i})$ and added noise *Z*, as follows:

$$\begin{array}{c} F_{i}=f_{i}\left(\mathrm{Pa}\left(F_{i}\right)\right)+Z. \end{array}$$

It is worth mentioning that the added noise *Z* is independent of $Pa(F_{i})$ and depends on the sensitivity of the deterministic function $f_{i}\left(\cdot \right)$. Because traditional score-based algorithms impose combinatorial acyclicity constraints while learning DAG from observational data, the estimation process becomes NP-hard [27]. To address this, the non-private FCM-based NOTEARS algorithm [17], introduces a continuous optimization problem which optimizes a score function score(W) as follows:

(4) $$\begin{array}{cc} \; & \min_{W\in R^{dxd}}\mathrm{score}\left(W\right)\;\mathrm{subject}\;\mathrm{to}\;h\left(W\right)=0 \end{array}$$

where the score function $\mathrm{score}\left(\cdot \right):R^{dxd}\to R$ is the combination of the squared loss function and a penalization function. Briefly, the score function is defined as

(5) $$\begin{array}{cc} \mathrm{score}(W,\alpha ) & =\underset{\mathrm{objective}\;\mathrm{function}}{\underset{︸}{\ell \left(W;D\right)+{\lambda ||W||}_{1}}}+\underset{\mathrm{quadratic}\;\mathrm{penalty}}{\underset{︸}{\frac{\rho }{2}{\left|h\left(W\right)\right|}^{2}}}+\underset{\mathrm{Lagrangian}\;\mathrm{multiplier}}{\underset{︸}{\alpha h(W)}}, \end{array}$$

where ρ>0 is a penalty parameter, α is the Lagrange multiplier, and ${\lambda ||W||}_{1}$ is a non-smooth penalizing term for dense graphs. The algorithm imposes the acyclicity constraint with $h:R^{dxd}\to R$, where h(·) is a smooth function over real matrices [17]. The acyclicity constraint is defined by the function h(W) as

$$\begin{array}{c} h\left(W\right)=\mathrm{tr}\left(e^{W\circ W}\right)-d=0, \end{array}$$

where ∘ is the *Hadamard product* and $e^{W\circ W}$ is the *matrix exponential* of W∘W. The acyclicity constraint h(W) is a non-convex function and has a gradient $\nabla h\left(W\right)={\left(e^{W\circ W}\right)}^{T}\circ 2W$ [17]. For a given dataset $D\in R^{nxd}$ with *n* i.i.d. samples of feature vector $F=(F_{1},\ldots ,F_{d})$, let D denote a discrete space of DAGs G=(V,E) on *d* nodes. The objective of the NOTEARS algorithm [17] is to model $\left(F_{1},\ldots ,F_{d}\right)$ via FCM. The $j^{th}$ feature is defined by $F_{j}=w_{j}^{T}F+Z$, where $F=(F_{1},\ldots ,F_{d})$ is a feature vector and $Z=(z_{1},\ldots ,z_{d})$ is an added noise vector.

***Differentially Private Score-Based CGD Algorithms:*** The optimization problem mentioned in (5) is non-private; therefore, releasing the gradient of the optimization problem is prone to privacy leakage. To address this privacy concern, the DP-preserving score-based CGD algorithm NOLEAKS [22] adopts the notion of differential privacy (DP) in the optimization process. To ensure differential privacy for the released gradient $\left(\hat{\nabla F}\right)$, the Jacobian of this optimization process is clipped with a certain clipping threshold (s) and perturbed with Gaussian noise $N(0,\sigma^{2}I_{\mathrm{dxd}})$.

Unlike constraint-based CGD algorithms, the later iterations are more critical than the initial ones when minimizing the score function score(W,α). The optimization process in score-based CGD algorithms uses gradient-based methods such as stochastic gradient descent (SGD). In differentially private SGD (DP-SGD), the privacy budget $\epsilon_{\mathrm{Total}}$, DP failure probabilities $\delta ,\delta^{\prime }$, and step size η are key factors that influence the path to finding the minima. Given a fixed privacy budget, adding the same amount of noise at each step can cause oscillations near the local minima; thus, introducing more noise in the initial iterations and gradually reducing it in subsequent iterations helps to achieve faster convergence. Therefore, initial iterations of the optimization process may handle more noise; however, as the algorithm tends to converge to the optima, the amount of added noise needs to be reduced for better convergence. This adaptivity in terms of added noise also ensures less chance of missing the optima. Motivated by this crucial fact, we introduce adaptivity to this setting and describe our proposed framework in the next section. As the NOLEAKS algorithm perturbs the Jacobian matrix through the *Gaussian noise* with the *same noise parameter (privacy budget)* to guarantee DP, the main difference between the existing NOLEAKS differential privacy framework and our proposed score-based *CURATE* framework is the per-iteration adaptive privacy budget increment during the perturbation of the Jacobian matrix.

***Adaptive Privacy Budgeting for Score-Based Algorithms:*** We observe some room for improvement in terms of adaptive privacy budget allocation for differentially private FCM-based CGD algorithms. Intuitively, the later steps/iterations in the optimization of (5) are more crucial compared to the initial ones, as the later iterations are closer to the optima. Recent works, including [33,34,35], have proposed adaptive privacy budget allocation mechanisms for gradient-based optimization problems that adaptively allocate privacy budgets for each iteration in the optimization process. In our proposed score-based framework, we aim to implement adaptive privacy budget allocation for each iteration and increment the privacy budget as a function of the iterations. Therefore, our goal is to select an adaptive privacy budgeting mechanism for score-based algorithms that allocates less privacy budget to the initial iterations compared to the later ones. Intuitively, privacy budgets can be incremented additively, multiplicatively, or exponentially. Next, we analyze these three different methods of incrementing the privacy budget as functions of the initial privacy budget $\epsilon_{0}$ and number of iterations *i*, and present some experimental results to highlight the method that achieves better F1-score.

In this paper, we analyze the performance of three different privacy budget increment mechanisms. Next, we brieflydemonstrate these mechanisms. First, in the *additive increment $\epsilon_{i}=\epsilon_{0}\left(1+\frac{i}{I}\right)$* scheme, the privacy budget of the $i^{th}$ iteration is defined as a linear function of the initial budget $\epsilon_{0}$, current iteration *i*, and total number of iterations *I*. Next, in the *exponential increment $\epsilon_{i}=\epsilon_{0}\times \exp\left(\frac{i}{I}\right)$* scheme, the budget of the $i^{th}$ iteration is incremented as a function of $\exp(\frac{i}{I})$. Finally, the *multiplicative increment $\epsilon_{i}=\epsilon_{0}^{\left(1+\frac{i}{I}\right)}$* scheme increments $\epsilon_{i}$ multiplicatively as a function of $\epsilon_{0}^{\frac{i}{I}}$.

**Lemma** **2.** *Given a total privacy budget of $\epsilon_{\mathrm{Total}}$ and initial privacy budget $\epsilon_{0}$, it is possible to execute a total possible number of iterations $I_{\mathrm{add}}=\frac{\epsilon_{\mathrm{Total}}+\frac{\epsilon_{0}}{2}}{\epsilon_{0}+\frac{\epsilon_{0}}{2}}$ (with additive increment), $I_{\exp}=\frac{\epsilon_{\mathrm{Total}}}{\epsilon_{0}\times \exp\left(1\right)}$ (with exponential increment), and $I_{\mathrm{mul}}=\frac{\log(\epsilon_{0})}{\log\left(1-\frac{\epsilon_{0}\left(1-\epsilon_{0}\right)}{\epsilon_{\mathrm{Total}}}\right)}$ for $\epsilon_{0}<1$ and $I_{\mathrm{mul}}=\frac{\log(\epsilon_{0})}{\log\left(1+\frac{\epsilon_{0}\left(\epsilon_{0}-1\right)}{\epsilon_{\mathrm{Total}}}\right)}$ for $\epsilon_{0}>1$ (with multiplicative increment).*

**Remark** **1.** *Combining the multiplicative and additive increments can be used to improve the number of iterations for different privacy regimes based on the total privacy budget and exhausted privacy budget.*

**Figure 4:**
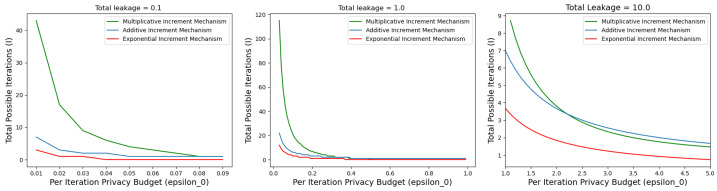
Possible number of iterations *I* given a total amount of privacy budget $\epsilon_{\mathrm{Total}}$ and initial privacy budget $\epsilon_{0}$. For varied total privacy budget $\epsilon_{\mathrm{Total}}=0.1,\epsilon_{\mathrm{Total}}=1.0$,$\epsilon_{\mathrm{Total}}=10.0$ and different initial budget $\epsilon_{0}<<1.0$ and $\epsilon_{0}>1.0$, it can be observed that the multiplicative method executes more iterations in the high-privacy regime (i.e., $\epsilon_{0}<<1.0$).

Lemma 2 shows explicit dependence of the total number of possible iterations on the total privacy budget $\epsilon_{\mathrm{Total}}$) and initial privacy budget $\epsilon_{0}$. The privacy budget incrementing methods can be readily adopted with the exponential mechanism, Laplace mechanism, and sparse vector technique. Figure 4 shows the maximum possible number of iterations with different adaptive methods given a fixed initial privacy budget $\epsilon_{0}$ and total privacy budget $\epsilon_{\mathrm{Total}}$. We also observe that the multiplicative method executes noticeably more iterations compared to the additive and exponential methods in higher-privacy regimes (i.e., $\epsilon_{0}<1$). The number of iterations directly influences the performance of the optimization process, as it follows a step-wise gradient-based method. Based on the total privacy budget $\epsilon_{\mathrm{Total}}$, if the process terminates before reaching the optimum, the algorithm will suffer from suboptimal performance. Therefore, the goal is to run as many iterations as possible based on the total and initial privacy budget. As we aim to achieve better performance by executing more iterations given a total privacy budget $\epsilon_{\mathrm{Total}}$, in this paper we follow the multiplicative method for incrementing the per-iteration privacy budget.

***Score-based CURATE Algorithm:*** Next, we present the adaptive private minimization technique used in score-based *CURATE* in Algorithm 2.
**Algorithm 2:** Adaptive Priv-Minimize
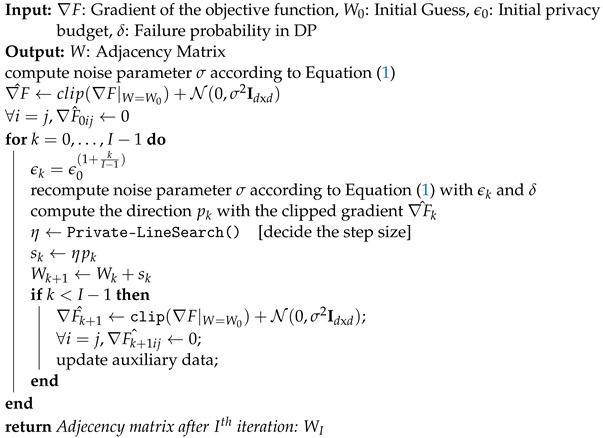


The function *clip(·)* denotes the clipping of the true gradient ∇F using a clipping threshold *s*, and can be mathematically represented as $\hat{\nabla F}=\nabla F/\max\left(1,\frac{{\left||\nabla F|\right|}_{2}}{s}\right)$ [32]. Clipping the gradient ensures that the gradient is bounded by the clipping threshold *s*. We use the Priv-Linesearch feature adopted from the NOLEAKS algorithm [22], by which the algorithm aims to investigate the optimal step size η. The score-based *CURATE* algorithm essentially utilizes the FCM-based model for CGD and allows for adaptive privacy budgeting through the optimization process. The score-based *CURATE* algorithm follows a similar FCM-based framework to the non-private NOTEARS algorithm and differentially private NOLEAKS algorithm; however, our proposed framework enables adaptive privacy budget allocation for each iteration through the Adaptive Priv-Minimize function.

***Remarks on the Score-Based CURATE Algorithm:*** Because the score-based *CURATE* algorithm follows an FCM-based workflow that is similar to the non-private NOTEARS and differentially private NOLEAKS algorithms, it achieves polynomial complexity in terms of the feature/variable size *d*. For small datasets and with less leakage, it achieves better and more meaningful causal graphs compared to constraint-based algorithms. However, due to the non-convex nature of the optimization problem, similar to NOTEARS and NOLEAKS algorithms, the score-based *CURATE* algorithm does not guarantee convergence to global optima. Nonetheless, we observed in our experiments that the score-based algorithms provided better privacy guarantees in regimes with lower total privacy ($\epsilon_{\mathrm{Total}}\leq 1$) compared to the differentially private constraint-based algorithms.

## 4. Results and Discussion

**Figure 5:**
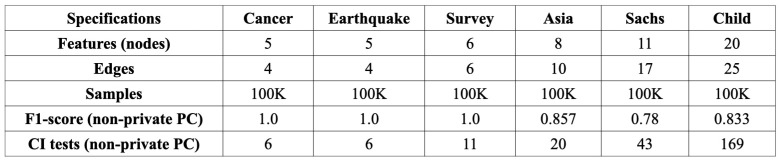
Dataset description and CGD results for the non-private PC algorithm [1] on six public CGD datasets with Kendall’s τ CI test statistic. The results were obtained with the following parameters: subsampling rate =1.0, test threshold =0.05).

***Data Description and Test Parameters:*** We compared the predictive performance of our proposed *CURATE* framework with non-private PC [1], EM-PC [21], SVT-PC, Priv-PC [20], and NOLEAKS [22] on six public CGD datasets [2,36,37,38,39]. Figure 5 presents the detailed description of the datasets along with the predictive performance of the non-private PC algorithm. For the experimental results, we considered the probability of failure in differential privacy $\delta^{\prime }=10^{-12}$, as the safe choice for $\delta^{\prime }$ is $\delta^{\prime }\leq n^{-1.5}$, where *n* is the total number of participants/samples in the dataset. In each of the six CGD datasets, the total number of samples was $\left(n\right)=100k=10^{5}$; thus, we considered the value of $\delta^{\prime }=10^{-12}\leq n^{-1.5}$. The total privacy budget ranged from $10^{-2}$ to $10^{2}$, and we varied the total budget in this range to observed the performance of the CGD algorithms in high-, moderate-, and low-privacy regimes. The initial privacy parameter was calculated based on Equation (3). The test threshold (T) was set as 0.05, the subsampling rate (q) was 1.0, and we used Kendall’s τ as a CI testing function for the constraint-based private algorithms. To run the experiments, we used a high-performance computing (HPC) system with one node and one CPU with 5 GB of RAM. The code for the constraint-based and score-based CURATE algorithm is available at https://github.com/PayelBhattacharjee14/cgdCURATE, accessed on 8 September 2024.

***Evaluation Metric:*** For the scope of our experiments, we measured the predictive performance of the CGD algorithms in terms of the F1-score, which indicates the similarity between the estimated graph G and ground truth $G^{*}$. Let the ground truth be represented by the graph $G^{*}=\left(V,E^{*}\right)$ and the estimated graph be represented by G=(V,E). The edges in the true graph are denoted as $E^{*}$ and the edges in the estimated graph are denoted as E. We can denote the precision as $\frac{E\cap E^{*}}{E}$ and recall as $\frac{E\cap E^{*}}{E^{*}}$; then, the F1-score (utility) of the CGD algorithm is defined as

$$F1=\frac{2\times \mathrm{Precision}\times \mathrm{Recall}}{\mathrm{Precision}+\mathrm{Recall}}.$$

***Privacy vs. Utility Tradeoff:*** There is a privacy–utility tradeoff in differential privacy-preserving CGD. Through comprehensive experimental results on six public CGD datasets, we observed that the private algorithms required higher privacy leakage to achieve the same predictive performance as their non-private counterparts.

**Figure 6:**
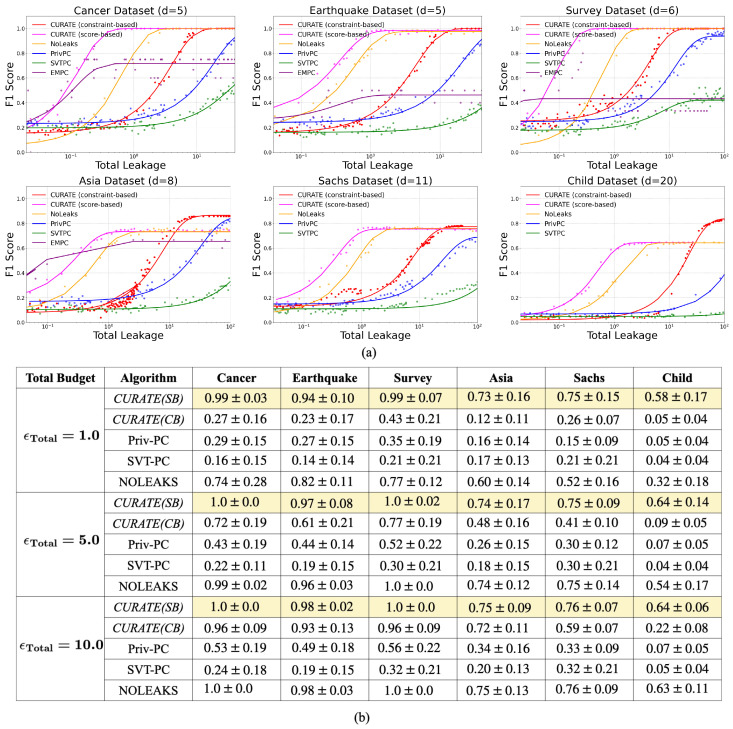
Part (**a**) presents the performance evaluation results of the differentially private CGD algorithms (EM-PC [21], SVT-PC, Priv-PC [20], NOLEAKS [22], and both score-based and constraint-based *CURATE*) in terms of total leakage vs. F1 score on six public CGD datasets: Cancer, Earthquake, Survey, Asia, Sachs, and Child. Part (**b**) presents the mean and standard deviation of the F1-score for 50 consecutive runs and for three privacy regimes ($\epsilon_{\mathrm{Total}}=0.1$, $\epsilon_{\mathrm{Total}}=5.0$, $\epsilon_{\mathrm{Total}}=10.0$).

The experimental results presented in Figure 6 show that with adaptive privacy budget allocation and minimization of total probability of error, *CURATE* outperforms the existing private CGD algorithms, including EM-PC [21], SVT-PC, Priv-PC [20], and NOLEAKS [22]. Figure 6 presents the mean F1-score and its standard deviation for 50 consecutive runs on the Cancer, Earthquake, Survey, Asia, Sachs, and Child datasets for different privacy regimes. The number of features in the dataset also impacts the performance of the CGD algorithms. Notably, for the Cancer, Earthquake, and Survey datasets, score-based *CURATE* achieves the highest F1-score with a total leakage of less than 1.0. As the number of features increases, *CURATE* and the other CGD algorithms tend to leak more in order to achieve the best F1-score. For the Sachs and Child datasets, *CURATE* achieves the highest F1-score with $\epsilon_{\mathrm{Total}}>1.0$. In addition, we observe that constraint-based *CURATE* achieves better utility (F1-score) with less total leakage compared to the existing constraint-based DP-CGD algorithms, including EM-PC [17], Priv-PC, and SVT-PC [20]. Therefore, adaptive privacy budgeting scales up utility in DP-CGD.

***Computational Complexity of DP-CGD Algorithms:*** The reliability of an algorithm also depends on its computational complexity. In private CGD, score-based and constraint-based algorithms have different computational complexities. As mentioned by the authors of [8], score-based algorithms are computationally expensive because they need to enumerate and assign scores to each possible output graph. For instance, NOLEAKS uses the *quasi-Newton*, method which has high computational and space complexity [22]; on the other hand, EM-PC is computationally slow, as the utility function used in the *exponential mechanism* is computationally expensive [20]. Priv-PC adopts SVT and the *Laplace mechanism* to ensure DP, whereas constraint-based *CURATE* optimizes privacy budgets $\bar{\epsilon }$ in an online setting and then adopts the *Laplace mechanism* to privatize CI tests. This makes *CURATE* less computationally expensive compared to the existing constraint-based DP-CGD algorithms.

**Figure 7:**
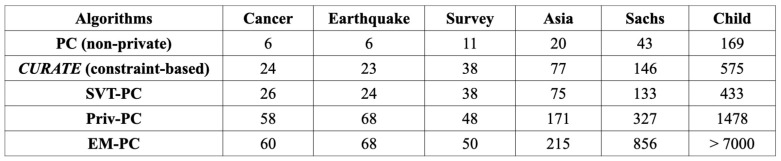
Average number of CI tests needed to achieve the maximum F1-score with a comparatively large amount of total leakage ($\epsilon_{\mathrm{Total}}=1.0$) on the Cancer, Earthquake, Survey, Asia, Sachs, and Child datasets. The average CI tests of *CURATE* converges to that of the non-private PC algorithm, whereas EM-PC [17], Priv-PC, and SVT-PC [20] tend to run more CI tests.

***Comparison of Number of CI Tests:*** The total number of CI tests executed by a differentially private CDG algorithm directly affects the privacy and utility tradeoff of the algorithm. The total number of CI tests in private constraint-based CGD algorithms directly influences the total amount of leakage, as each CI test is associated with some amount of privacy leakage. Privacy leakage can be provably reduced by efficient and accurate CI testing. In the constraint-based *CURATE* algorithm, privacy budgets are allocated by minimizing a surrogate for the total probability of the error. Intuitively, this decreases the total leakage of *CURATE*, as the adaptive choice of privacy budgets makes the initial CI tests more accurate. Therefore, *CURATE* tends to run a smaller number of CI tests compared to other differentially private algorithms. We confirm this intuition in the results presented in Figure 7. It can be observed that the number of CI tests in EM-PC, SVT-PC, and Priv-PC are comparatively large relative to *CURATE* and the non-private counterpart PC algorithm [1].

**Figure 8:**
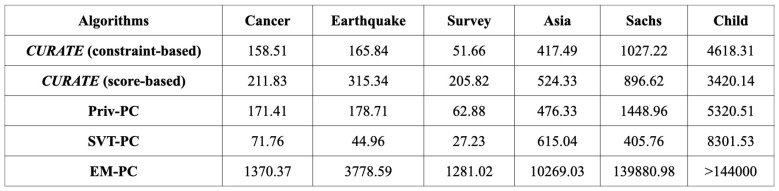
Running time comparison (in seconds) of differentially private constraint-based and score-based algorithms on six public CGD datasets: Cancer, Earthquake, Survey, Asia, Sachs, and Child for 50 consecutive iterations.

***Running Time Comparison:*** In this subsection of the paper, we provide a running time comparison between adaptive and non-adaptive score-based and constraint-based differentially private CGD algorithms. Due to their complexity, score-based CGD algorithms tend to consume more time compared to constraint-based algorithms. Figure 8 compares the running times of the differentially private CGD algorithms for 50 consecutive iterations. As shown in the figure, the constraint-based *CURATE* algorithm speeds up the process of DP-CGD compared to the Priv-PC and EM-PC algorithms, while the score-based *CURATE* algorithm achieves better predictive performance compared to the NOLEAKS algorithm with a similar amount of execution time. The adaptivity of these DP-CGD algorithms allows them to converge faster and reduces their overall execution time.

## 5. Conclusions

This paper proposes *CURATE*, a differentially private causal graph discovery framework that scales up privacy via adaptive privacy budget allocation for both constraint-based and score-based CGD environments. Constraint-based *CURATE* is based on the key idea of minimizing a surrogate for the total probability of error in CGD, which ensures a better privacy–utility tradeoff. Score-based *CURATE* allows a higher number of iterations and faster convergence of the optimization problem through adaptive budgeting, thereby guaranteeing better utility with less leakage. In our experiments, we observed that the average number of CI tests required with constraint-based *CURATE* is similar to the number of CI tests required by the non-private PC algorithm. Our results show that *CURATE* outperforms existing private CGD algorithms and achieves better utility. In addition, the leakage of the proposed framework using adaptive privacy budgeting is smaller by orders of magnitude. In addition, there are several interesting open research directions for future work: (i) an adaptive gradient-clipping mechanism could be implemented for the score-based algorithm; (ii) as our proposed score-based framework uses the resulting pruned graph, the per-iteration privacy budget could be designed based on the outcome of the previous iteration; and (iii) the outcomes of previous noisy tests could be used to tune hyperparameters such as the test threshold, margins, and clipping threshold.

## 6. Remarks

A part of this work, our constraint-based *CURATE* algorithm has been submitted and accepted to the 2024 IEEE International Workshop on Machine Learning for Signal Processing (IEEE MLSP 2024). This article is a revised and expanded version of a paper titled Adaptive Privacy for Differentially Private Causal Graph Discovery which we presented at the IEEE MLSP 2024 conference in London, UK, on 25 September 2024.

## Data Availability

We have used publicly available datasets for our experiments, and they are cited within the article. The code and used datasets are also available in CURATE GitHub repository as mentioned in the Experimental Results section.

## Appendix A

### Appendix A.1. Proof of Lemma 1

In this section, we present the proof of Lemma 1. For every order-*i* conditional independence (CI) test, we have a privacy budget of $\epsilon_{i}$. Given a CI test statistic f(D) with $l_{1}$-sensitivity $\Delta_{1}$, threshold *T*, and margins $\left(\beta_{1},\beta_{2}\right)$, we perturb the test statistic using *Laplace noise*, defined as $Z=Lap(\frac{\Delta_{1}}{\epsilon_{i}})$, and check for conditional independence between $(v_{a},v_{b})\in G$ conditioned on *S* as follows:

1. If $f\left(D\right)+Z>T\left(1+\beta_{2}\right)\Rightarrow$ delete edge $\left(v_{a},v_{b}\right)$
2. If $f\left(D\right)+Z<T\left(1-\beta_{1}\right)\Rightarrow$ keep edge $\left(v_{a},v_{b}\right)$
3. Else keep edge ($v_{a},v_{b}$) with probability $\frac{1}{2}$.

For simplicity of notation, we define $f_{v_{a},v_{b}|S}\left(D\right):=f\left(D\right)$.

**Type-I Error:** We now analyze the type-I error relative to the unperturbed CI test, i.e., when the private algorithm keeps the edge given that the unperturbed test statistic deletes the edge f(D)>T. In other words, this can be written as $P\left(E_{1}^{i}\right)=P\left(\mathrm{Error}|f\left(D\right)>T\right)$. We note that the error event occurs only for cases (b) and (c). We can bound the relative type-I error as follows:

(A1) $$\begin{array}{cc} \; & P\left(E_{1}^{i}\right)=P\left(\mathrm{Error}|f\left(D\right)>T\right)\\ \; & \leq \frac{1}{2}\left(P(f\left(D\right)+Z\in \left[T\left(1-\beta_{1}\right),T\left(1+\beta_{2}\right)\right]|f\left(D\right)>T)\right) \end{array}$$

(A2) $$\begin{array}{cc} \; & \;+P(f\left(D\right)+Z<T\left(1-\beta_{1}\right)|f\left(D\right)>T) \end{array}$$

(A3) $$\begin{array}{cc} \; & \leq \frac{c_{1}}{2}+P\left(f\left(D\right)+Z<T\left(1-\beta_{1}\right)|f\left(D\right)>T\right)\leq \frac{c_{1}}{2}+\frac{1}{2}\exp\left(\frac{-T\beta_{1}\epsilon_{i}}{\Delta_{1}}\right) \end{array}$$

where the last inequality follows from the Laplacian tail bound and the fact that f(D)>T, and we have defined $c_{1}$ as $c_{1}:=P\left(f\left(D\right)+Z\in \left[T\left(1-\beta_{1}\right),T\left(1+\beta_{2}\right)\right]|f\left(D\right)>T\right)$.

The upper bound on $P[f\left(D\right)+Z<T\left(1-\beta_{1}\right)|f\left(D\right)>T]$ is obtained from the *Laplace tail bound* as follows:

(A4) $$\begin{array}{cc} \; & P\left[f\left(D\right)+Z<T\left(1-\beta_{1}\right)|f\left(D\right)>T\right]=P\left[Z<T\left(1-\beta_{1}\right)-f\left(D\right)\right]\\ \; & =\frac{1}{2}\exp\left(\frac{T-T\beta_{1}-f\left(D\right)}{\Delta_{1}/\epsilon_{i}}\right)\leq \frac{1}{2}\exp\left(\frac{T-T\beta_{1}-T}{\Delta_{1}/\epsilon_{i}}\right)\\ \; & =\frac{1}{2}\exp\left(\frac{-T\beta_{1}\epsilon_{i}}{\Delta_{1}}\right). \end{array}$$

**Type-II Error:** Next, we analyze the type-II error relative to the unperturbed CI test, i.e., when the differentially private algorithm deletes an edge given that the unperturbed CI test statistic keeps the edge (f(D)<T). Mathematically, $P\left[E_{2}^{i}\right]=P\left(\mathrm{Error}|f\left(D\right)<T\right).$ The type-II error occurs only for cases (a) and (c). Therefore, we can bound the type-II error as follows:

(A5) $$\begin{array}{cc} \; & P\left(E_{2}^{i}\right)=P\left(\mathrm{Error}|f\left(D\right)<T\right)\\ \; & \leq \frac{1}{2}\left(P(f\left(D\right)+Z\in \left[T\left(1-\beta_{1}\right),T\left(1+\beta_{2}\right)\right]|f\left(D\right)<T)\right) \end{array}$$

(A6) $$\begin{array}{cc} \; & \;+P(f\left(D\right)+Z>T\left(1+\beta_{2}\right)|f\left(D\right)<T) \end{array}$$

(A7) $$\begin{array}{cc} \; & \leq \frac{c_{2}}{2}+P\left(f\left(D\right)+Z>T\left(1+\beta_{2}\right)|f\left(D\right)<T\right) \end{array}$$

(A8) $$\begin{array}{cc} \; & \leq \frac{c_{2}}{2}+\frac{1}{2}\exp\left(\frac{-T\beta_{2}\epsilon_{i}}{\Delta_{1}}\right) \end{array}$$

where the last inequality follows from the Laplacian tail bound and the fact that f(D)<T, and we have defined $c_{2}$ as $c_{2}:=P\left(f\left(D\right)+Z\in \left[T\left(1-\beta_{1}\right),T\left(1+\beta_{2}\right)\right]|f\left(D\right)<T\right)$. The probability $P[f\left(D\right)+Z>T\left(1+\beta_{2}\right)|f\left(D\right)<T]$ can also be upper bounded as

$$\begin{array}{cc} P[f(D) & +Z>T\left(1+\beta_{2}\right)\left|f\left(D\right)<T\right]=P\left[Z>T\left(1+\beta_{2}\right)-f\left(D\right)\right] \end{array}$$

(A9) $$\begin{array}{cc} \; & =\frac{1}{2}\exp\left(-\frac{T+T\beta_{2}-f\left(D\right)}{\Delta_{1}/\epsilon_{i}}\right)\leq \frac{1}{2}\exp\left(-\frac{T+T\beta_{2}-T}{\Delta_{1}/\epsilon_{i}}\right) \end{array}$$

(A10) $$\begin{array}{cc} \; & =\frac{1}{2}\exp\left(\frac{-T\beta_{2}\epsilon_{i}}{\Delta_{1}}\right). \end{array}$$

This concludes the proof of Lemma 1.

### Appendix A.2. Sensitivity Analysis of Weighted Kendall’s τ

Conditional independence (CI) tests in causal graph discovery (CGD) measure the dependence of one variable ($v_{a}$) on another ($v_{b}$) conditioned on a set of variables. Let the CI test statistic for connected variable pairs ($v_{a},v_{b}$) in graph G be τ(D) for dataset D and $\tau (D^{\prime })$ for dataset $D^{\prime }$. For large samples, the test statistic τ(·) follows a *Gaussian distribution*. Therefore, the sensitivity can be defined as follows:

(A11) $$\begin{array}{cc} \Delta_{1}\left(\Phi \left(\tau (D)\right)\right) & =\underset{D\neq D^{\prime }}{\sup}\left|\Phi \left(\tau (D)\right)-\Phi \left(\tau (D^{\prime })\right)\right|\leq \Delta \left(\Phi \left(\cdot \right)\right)\times \Delta \left(\tau (\cdot )\right)\\ \; & =\underset{D\neq D^{\prime }}{\sup}\frac{|\Phi \left(\tau (D)\right)-\Phi \left(\tau (D^{\prime })\right)|}{|\tau (D)-\tau (D^{\prime })|}\times \left|\tau (D)-\tau (D^{\prime })\right|\\ \; & \leq L_{\Phi }\times \sup\left|\tau (D)-\tau (D^{\prime })\right| \end{array}$$

where $\sup_{D\neq D^{\prime }}\left|\tau \left(D\right)-\tau \left(D^{\prime }\right)\right|$ is the $l_{1}$-sensitivity of the CI test statistic for datasets D and $D^{\prime }$, with Φ as the PDF of the standard normal distribution. Because Φ(·) is differentiable, the Lipschitz constant ($L_{\Phi }$) can be upper bounded as $L_{\Phi }\leq \frac{1}{\sqrt{2\pi }}$. Thus, the sensitivity can easily be calculated using the sensitivity of the weighted test statistic.

**$l_{1}$-Sensitivity Analysis:** For large sample sizes (n>>1), Kendall’s τ test statistic follows a Gaussian distribution with zero mean and variance $\frac{2(2n+5)}{9n(n-1)}$, where *n* is the number of i.i.d. samples. Given a dataset D with *d* features, the conditional dependence of between variables ($v_{a},v_{b}$) conditioned on set *S* can be measured with Kendall’s τ as a CI test statistic. For instance, the data can be split into *k* bins according to the unique values of set *S*. For each $i^{th}$ bin, the test statistic $\tau_{i}$ is calculated; the weighted average of all $\tau_{i}$ values represents the test statistic for the entire dataset. The weighted average [20] is defined as $\tau =\frac{\sum_{i=1}^{k}w_{i}\tau_{i}}{\sqrt{\sum_{i=1}^{k}w_{i}}}$, where $w_{i}$ is the inverse of the variance $w_{i}=\frac{9n_{i}\left(n_{i}-1\right)}{2(2n_{i}+5)}$.

As we perturb the *p*-value obtained from this weighted test statistic, we need to observe the $l_{1}$-sensitivity of *p*-value. For the scope of this paper, we consider the *Lipschitz constant* of Gaussian distribution while calculating the sensitivity.

The weighted average τ essentially follows the standard normal distribution, i.e., τ∼N(0,1). Hence, the $l_{1}$-sensitivity of *p*-value can be defined as

(A12) $$\begin{array}{cc} \Delta_{1} & =|\Phi (\tau \left(D\right)-\Phi (\tau \left(D^{\prime }\right)|=\frac{|\Phi (\tau \left(D\right)-\Phi (\tau \left(D^{\prime }\right)|}{|\tau \left(D\right)-\tau \left(D^{\prime }\right)|}\times \left|\tau \left(D\right)-\tau \left(D^{\prime }\right)\right|\\ \; & \leq L_{\Phi }|\tau \left(D\right)-\tau \left(D^{\prime }\right)|\leq \frac{1}{\sqrt{2\pi }}\left|\tau \left(D\right)-\tau \left(D^{\prime }\right)\right|. \end{array}$$

The sensitivity of this weighted Kendall’s τ can be expressed as

$$\begin{array}{cc} \Delta_{1}\left(\tau \right) & =\max_{|D^{\prime }-D|\leq 1}\left|\tau \left(D^{\prime }\right)-\tau \left(D\right)\right|\leq \Delta_{1}\left(\tau_{i}\right)\Delta_{1}\left(w_{i}\right). \end{array}$$

The sensitivity of $\tau_{i}$ depends upon the number of elements $n_{i}$ and $\Delta_{1}\left(\tau_{i}\right)\leq \frac{2}{n_{i}-1}$ [20]. The sensitivity of weights $\Delta (w_{i})$ can be represented as follows:

(A13) $$\begin{array}{cc} \Delta_{1}\left(w_{i}\right) & \leq \left|\frac{w_{i}^{\prime }}{\sqrt{\sum_{i\neq j}^{k}w_{j}+w_{i}^{\prime }}}-\frac{w_{i}}{\sqrt{\sum_{j=1}^{k}w_{j}}}\right|\leq \left|\frac{\frac{9n_{i}\left(n_{i}+1\right)}{2(2\left(n_{i}+1\right)+5)}}{\sqrt{\sum_{j=1}^{k}w_{j}+w_{i}^{\prime }}}\right|-\left|\frac{\frac{9n_{i}\left(n_{i}-1\right)}{2(2n_{i}+5)}}{\sqrt{\sum_{j=1}^{k}}w_{j}}\right|. \end{array}$$

We can provide an upper bound on Equation (A13) through the triangle inequality, and the sensitivity of the weight can be bounded as

(A14) $$\begin{array}{c} \Delta_{1}\left(w_{i}\right)\leq \sqrt{\frac{2}{n}}\left(\left|\frac{9n_{i}\left(n_{i}+1\right)}{2(2\left(n_{i}+1\right)+5)}\right|-\left|\frac{9n_{i}^{2}}{2(2n_{i}+5)}\right|\right). \end{array}$$

The sensitivity Δ(τ) essentially depends upon the number of elements in the $i^{th}$ bin (the bin that changes due to the addition or removal of a single user). For a dataset with a block size of at least size c and kc≈n, the overall sensitivity for the *p*-value can be bounded with Equations (A12) and (A14) as follows:

(A15) $$\begin{array}{cc} \Delta_{1} & \leq \frac{1}{\sqrt{2\pi }}\times \frac{2}{n_{i}-1}\times \sqrt{\frac{2}{n}}\times \left(\left|\frac{9n_{i}\left(n_{i}+1\right)}{2(2\left(n_{i}+1\right)+5)}\right|-\left|\frac{9n_{i}^{2}}{2(2n_{i}+5)}\right|\right)\\ \; & =\frac{2}{\sqrt{n\pi }}\left(\frac{\left|\frac{9n_{i}\left(n_{i}+1\right)}{2(2\left(n_{i}+1\right)+5)}\right|-\left|\frac{9n_{i}^{2}}{2(2n_{i}+5)}\right|}{n_{i}-1}\right). \end{array}$$

This concludes the $l_{1}$-sensitivity analysis of the weighted Kendall’s τ coefficient.

### Appendix A.3. Proof of Lemma 2

Finally, we analyze the methods adopted for the class of score-based algorithms. Below, we present the proof of Lemma 2. The main objective is to derive the relationship between the total privacy leakage $\epsilon_{\mathrm{Total}}$, number of iterations *I*, and the initial privacy budget $\epsilon_{0}$ for the score-based *CURATE* algorithm. We first demonstrate the possible number of iterations for the additive, multiplicative, and exponential incrementing methods for the score-based *CURATE* algorithm.

**Additive Incrementing Method:** This method increments the privacy budget for each iteration $\epsilon_{i}$ as a function of the current number of iterations *i*, total assigned privacy budget $\epsilon_{\mathrm{Total}}$, and initial privacy budget $\epsilon_{0}$. Mathematically, for every $i^{th}$ iteration, this method increments the privacy budget for each iteration as $\epsilon_{i}=\epsilon_{0}\left(1+\frac{i}{I_{\mathrm{add}}}\right)$. Given a total privacy budget $\epsilon_{\mathrm{Total}}$, initial privacy budget $\epsilon_{0}$, and number of iterations $I_{\mathrm{add}}$, we can define $\epsilon_{\mathrm{Total}}$ as

$$\begin{array}{cc} \; & \epsilon_{\mathrm{Total}}=\frac{I_{\mathrm{add}}}{2}\left[2\epsilon_{0}+\left(I_{\mathrm{add}}-1\right)\frac{\epsilon_{0}}{I_{\mathrm{add}}}\right]\\ \; & I_{\mathrm{add}}=\frac{\epsilon_{\mathrm{Total}}+\frac{\epsilon_{0}}{2}}{\epsilon_{0}+\frac{\epsilon_{0}}{2}}. \end{array}$$

**Exponential Incrementing Method:** This method increments the per-iteration privacy budget as an exponential function of the initial budget $\epsilon_{0}$ and current number of iterations *i*. For every $i^{th}$ iteration, the exponential incrementing method defines the privacy budget as $\epsilon_{i}=\epsilon_{0}\times \exp\left(\frac{i}{I_{\exp}}\right)$. Given a total privacy budget $\epsilon_{\mathrm{Total}}$, initial privacy budget $\epsilon_{0}$, and possible number of iterations $I_{\exp}$, we define $\epsilon_{\mathrm{Total}}$ as

$$\begin{array}{cc} \; & \exp\left(0\right)\leq \exp\left(1/I_{\exp}\right)\leq \ldots \leq \exp\left(I_{\exp}/I_{\exp}\right)\\ \; & \sum_{i=0}^{I_{\exp}}\exp\left(\frac{i}{I_{\exp}}\right)\leq I_{\exp}\exp\left(1\right). \end{array}$$

To maintain the total privacy budget of $\epsilon_{\mathrm{Total}}$, we can define the relationship between $I_{\exp},\;\epsilon_{\mathrm{Total}}$, and $\epsilon_{0}$ as

$$\begin{array}{cc} \; & \epsilon_{\mathrm{Total}}\geq I_{\exp}\times \exp\left(1\right)\times \epsilon_{0}\\ \; & I_{\exp}\leq \frac{\epsilon_{\mathrm{Total}}}{\epsilon_{0}\times \exp\left(1\right)}. \end{array}$$

**Multiplicative Incrementing Method:** This method enables the algorithm to increment the per-iteration privacy budget $\epsilon_{i}$ as a multiplicative function of the initial budget $\epsilon_{0}$ and the current number of iterations. For every $i^{th}$ iteration, the per-iteration privacy budget $\epsilon_{i}$ is defined as $\epsilon_{i}=\epsilon_{0}^{\left(1+\frac{i}{I_{\mathrm{mul}}}\right)}$. In this method, the possible number of iterations $I_{\mathrm{mul}}$ depends on the value of the factor $\epsilon_{0}^{1/I_{\mathrm{mul}}}$. If $\epsilon_{0}^{1/I_{\mathrm{mul}}}\leq 1$, then $\epsilon_{0}\leq 1$, which indicates a high-privacy regime; otherwise, there is a low-privacy regime where $\epsilon_{0}^{1/I_{\mathrm{mul}}}\geq 1$ and $\epsilon_{0}\geq 1$. For the high-privacy regime $\epsilon_{0}\leq 1$, we define the total leakage $\epsilon_{\mathrm{Total}}$ as

(A16) $$\begin{array}{cc} \epsilon_{\mathrm{Total}} & =\frac{\epsilon_{0}\left(1-\epsilon_{0}^{\frac{1}{I_{\mathrm{mul}}}\times I_{\mathrm{mul}}}\right)}{1-\epsilon_{0}^{1/I_{\mathrm{mul}}}}=\frac{\epsilon_{0}\left(1-\epsilon_{0}\right)}{1-\epsilon_{0}^{1/I_{\mathrm{mul}}}}\\ I_{\mathrm{mul}} & =\frac{\log(\epsilon_{0})}{\log\left(1-\frac{\epsilon_{0}\left(1-\epsilon_{0}\right)}{\epsilon_{\mathrm{Total}}}\right)}. \end{array}$$

For the case with initial privacy budget $\epsilon_{0}>1$, we can derive the expression of $I_{\mathrm{mul}}$ as follows:

(A17) $$\begin{array}{cc} \epsilon_{\mathrm{Total}} & =\frac{\epsilon_{0}\left(\epsilon_{0}^{\frac{1}{I_{\mathrm{mul}}}\times I_{\mathrm{mul}}}-1\right)}{\epsilon_{0}^{\frac{1}{I_{\mathrm{mul}}}}-1}=\frac{\epsilon_{0}\left(\epsilon_{0}-1\right)}{\epsilon_{0}^{\frac{1}{I_{\mathrm{mul}}}}-1}\\ I_{\mathrm{mul}} & =\frac{\log(\epsilon_{0})}{\log\left(\frac{\epsilon_{0}\left(\epsilon_{0}-1\right)}{\epsilon_{\mathrm{Total}}}+1\right)}. \end{array}$$

This concludes the proof of Lemma 2.
